# A different suite: The assemblage of distinct fungal communities in water-damaged units of a poorly-maintained public housing building

**DOI:** 10.1371/journal.pone.0213355

**Published:** 2019-03-18

**Authors:** Iman A. Sylvain, Rachel I. Adams, John W. Taylor

**Affiliations:** Department of Plant and Microbial Biology, University of California, Berkeley, Berkeley, California, United States of America; Wadsworth Center, UNITED STATES

## Abstract

Water-damaged housing has been associated with a number of negative health outcomes, principally respiratory disease and asthma. Much of what we know about fungi associated with water-damaged buildings has come from culture-based and immunochemical methods. Few studies have used high-throughput sequencing technologies to assess the impact of water-damage on microbial communities in residential buildings. In this study we used amplicon sequencing and quantitative-PCR to evaluate fungal communities on surfaces and in airborne dust in multiple units of a condemned public housing project located in the San Francisco Bay Area. We recruited 21 households to participate in this study and characterized their apartments as either a unit with visible mold or no visible mold. We sampled airborne fungi from dust settled over a month-long time period from the outdoors, in units with no visible mold, and units with visible mold. In units with visible mold we additionally sampled the visible fungal colonies from bathrooms, kitchens, bedrooms, and living rooms. We found that fungal biomass in settled dust was greater outdoors compared to indoors, but there was no significant difference of fungal biomass in units with visible mold and no visible mold. Interestingly, we found that fungal diversity was reduced in units with visible mold compared to units with no visible mold and the outdoors. Units with visible mold harbored fungal communities distinct from units with no visible mold and the outdoors. Units with visible mold had a greater abundance of taxa within the classes Eurotiomycetes, Saccharomycetes, and Wallemiomycetes. Colonies of fungi collected from units with visible mold were dominated by two *Cladosporium* species, *C*. *sphaerospermum* and *C halotolerans*. This study demonstrates that high-throughput sequencing of fungi indoors can be a useful strategy for distinguishing distinct microbial exposures in water-damaged homes with visible and nonvisible mold growth, and may provide a microbial means for identifying water damaged housing.

## Introduction

It is well established that humans spend most of their time in their homes; the estimate is 70% for residents of the United States [1]. It is equally well established that housing is a major social determinant of health [2]. Damp, moldy housing is linked with a number of negative health outcomes, such as respiratory infections, asthma, allergy, and compromised mental health [3]. Given that ethnic minorities and low-income populations disproportionately occupy inadequate and unhealthy housing, there is resultant public health disparity [4]. Acknowledging these factors, the World Health Organization recognizes access to a healthy indoor environment as a basic human right [5], and some public health practitioners view the desegregation of North American housing as an environmental justice priority [6].

The health of homes can be compromised by water intrusion through leaks (in roofs, windows, or plumbing), floods, or condensation, because increased moisture promotes the proliferation of microbes on indoor surfaces [7]. There has long been an effort to identify microbial, and specifically fungal, signatures of water damage in buildings, and to pinpoint the potential cause of ill-health effects associated with damp housing conditions [8]. These efforts have largely relied on assessment of microbial communities through cultivation and immunological techniques. Based on this work, many of the taxa that proliferate on water damaged building materials have been identified [9]. Some of these taxa are capable of producing mycotoxins [10], which creates an additional concern for public health. While some studies have found that concentrations of cultivable fungi or fungal cell wall components are higher in damaged houses compared to dry homes [11], this pattern has not always been observed [12].

Reliance on cultivation as part of the process to identify microbes is now known to be inadequate following the discovery that the vast majority of microorganisms are unculturable under laboratory conditions [13]. Instead, cultivation-free approaches provide a promising new approach to grant insight into the microbiology of the built environment [14], including water-damaged buildings. By utilizing DNA sequencing platforms such as Illumina MiSeq and 454 Pyrosequencing for samples of house dust, much has been learned about the diverse bacteria and fungi that co-occur in our dwellings [15]. It is now known that indoor microbial communities are structured by patterns of geography and climate [16], season [17], building design [18], ventilation systems [19], and occupants [20]. Fungal taxa found in house dust from healthy buildings have been shown largely to be a stochastic subset of outdoor fungi, presumably trafficked inside via open doors, windows, and on residents [21], and their presence is principally determined by the geographic location of the home [22].

Advances in sampling indoor air have also facilitated our greater understanding of the airborne mycobiome. Indoor samples typically fall into one of two types, either surface sampling using swabs, wipes, or tape, to recover microbes from suspected colonies, or vacuum sampling of either air or dust settled on floors and shelving [8]. Swab and tape sampling is useful for identifying colonies of visible fungi; air sampling provides a sample of airborne microbes at a defined time, typically a few minutes; and settled dust provides a sample integrated over a long, but undefined, period [23]. The adoption of a new approach to sampling airborne microbes by gravity settlement over a defined period of at least one-week [24] can account for diurnal variation in airborne microbes and variation in occupant behavior. Importantly, this approach avoids the expense of vacuum pumps and trained technicians, allowing for far greater replication and implementation by residents themselves.

Researchers are just beginning to apply high-throughput sequencing techniques to characterize fungal communities in water-damaged housing. Recently Jayaprakash et al. [25] applied amplicon sequencing to severely moisture-damaged residences undergoing renovation, complimented with quantitative-PCR (qPCR) and chemical-analytical approaches. Renovation of residences damaged by moisture resulted in a decrease in overall fungal richness and had a small but significant effect on fungal community composition. Prior to this, Emerson et al. [26] conducted amplicon sequencing and qPCR on house dust from passive dust collectors and HVAC filters in flooded and non-flooded homes six months after a historic weather event in Boulder, Colorado. They found significant differences in fungal community composition between flooded and non-flooded homes, with flooded homes hosting three times greater fungal biomass, and experiencing dominance by *Penicillium* taxa.

We have heeded the call to further study the relationship between the water-damaged built environment, microbial communities, and human occupants [27]. We have previously examined the processes that govern fungal community assemblage in healthy housing by sampling airborne dust in newly-constructed university housing in the San Francisco Bay Area [21]. Here we used the same sampling and identification approaches to examine fungal assemblages in poorly maintained, water-damaged residences in the Bay Area. We sampled from a public housing project that had been condemned by the local Housing Authority due to chronic disrepair. The building had long-term water damage including a leaking roof that had produced stalactites from dissolved concrete. The resulting water intrusion supported visible fungal colonies in units, and the building was additionally plagued with pest infestations. With this data, we asked whether broad differences in fungal community structure could be detected in units with visible mold when compared to units with no visible mold or the outdoors.

## Materials and methods

### Sampling

Fungi in air and on surfaces were sampled at a 6-story, 150-unit, concrete public housing project in the San Francisco Bay Area that was built in 1966. The building remained occupied with residents despite having been declared uninhabitable in 2014. Federal reports had documented the roof leaks that over two decades of disrepair led to the formation of stalactites, as well as pest infestations, sewage problems, asbestos, and exposed electrical wire.

At a Residents Council meeting, 21 households spanning the 6 stories in the building and inhabiting units with varied layouts volunteered to participate in this study. At the initial sampling visit, experienced mycologists surveyed the apartment and categorized the units (individual households within the building) as having either ‘visible mold’ growth or ‘no visible mold’. Visible mold was present in kitchens, bathrooms, livings, and bedrooms, and appeared as green, black, or pinkish-orange growths and discoloration on walls; the extent of the visible mold was not recorded. We categorized 11 units as having ‘visible mold’ and 10 units as having ‘no visible mold’. We noted the floor level of the unit and room where samples were collected for each sample.

**Fig 1:**
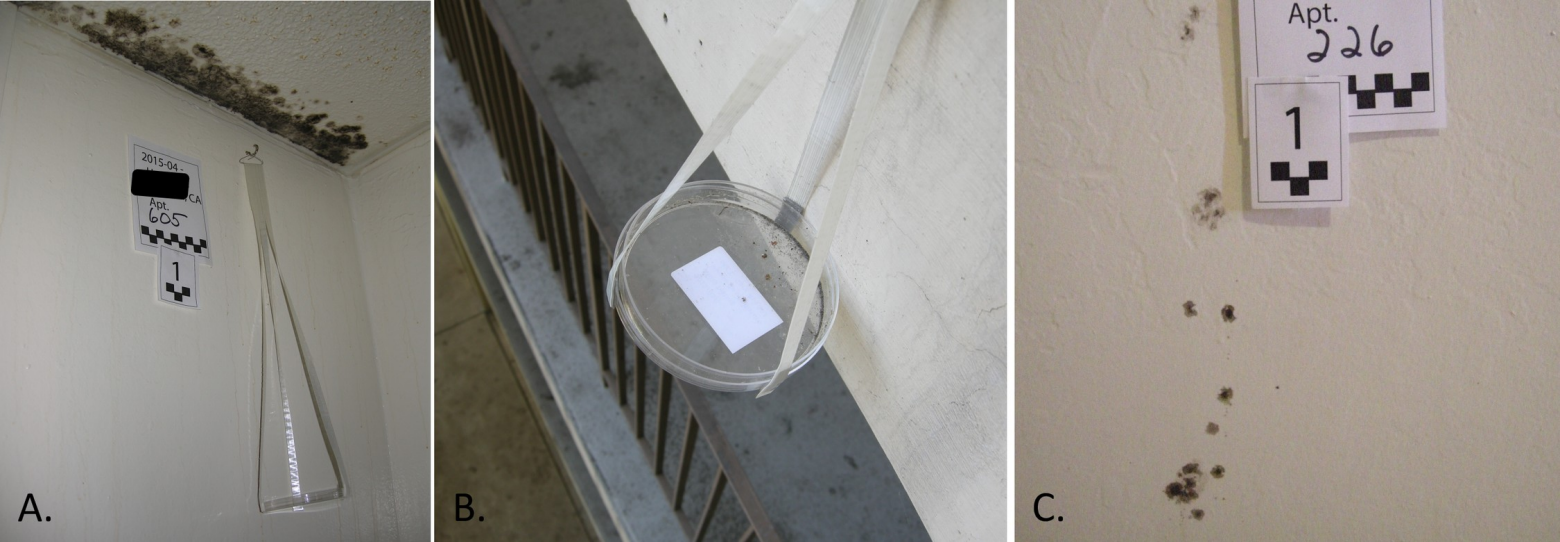
Photographs of sampling methods. Indoor (A) and outdoor (B) settled dust collectors. These empty, sterile petri dishes collect airborne fungi by settlement of dust over the course of four weeks. Patches of visible mold or obvious discoloration on walls were swabbed for surface samples (C).

Airborne house dust was collected from each unit using settled dust collectors (open, empty, sterile 10cm diameter petri dishes) [24], that were left open for four weeks in kitchens, bathrooms, living rooms, and bedrooms (Fig 1A). Indoor settled dust samples were paired with outdoor samples (Fig 1B). Outdoor samples were obtained from collectors suspended from railings or placed on top of light fixtures adjacent to the units and protected from precipitation by an overhang. In all units, settled dust collectors were placed in living rooms, bathrooms, and the outdoor walkway. In units with visible mold, additional settled dust collectors were also placed to sample all rooms with visible mold. In total, 68 settled dust samples were analyzed (18 outdoor samples, 22 indoors samples from units with no visible mold, and 28 indoor samples from units with visible mold). In units with visible mold, fungal colonies were sampled directly (Fig 1C) with dry Floq Swabs (Copan Diagnostics). These 24 surface samples were collected and frozen until processed. The study was approved by the University of California Committee for the Protection of Human Subjects under protocol 2014-08-6589.

### DNA extraction and library preparation

Fungal genomic DNA (gDNA) was extracted from settled dust samples using a phenol:choloform-isoamyl alcohol extraction protocol, followed by MoBio PowerSoil Kit, as previously described [21]. Surface samples (collected from units with visible mold) were processed following the PowerSoil Kit without modification. As controls, we also processed gDNA from unexposed swabs and petri dishes in order to determine potential contamination or sequencing errors in downstream analyses. We included a Mock Community sample composed of gDNA from 18 known taxa, including Rhodotorula, Cladosporium, Phoma, Candida in high relative concentrations; Cryptococcus, Candida, and two taxa of Penicillium in intermediate relative concentrations; and Stachybotrys, Neurospora, Chaetomium, Tetrasphaeria, Beauveria, Leptosphaerulina, Pestalotiopsis, Exophiala, and additional taxa of Cladosporium and Penicillium in low relative concentrations.

Fungal community composition was determined by constructing amplicon libraries from gDNA isolated from settled dust samples and swabbed colonies. Briefly, PCR primers for the ribosomal DNA internal transcribed spacer (ITS) regions, ITS1 and ITS2 [28] were adapted for Illumina MiSeq 250 paired-end sequencing with V2 chemistry, following methods previously described [29]. Quality of PCR amplicons was assessed by gel electrophoresis, prior to further cleaning with magnetic beads, and quantification using a Quant-iT dsDNA Assay Kit. Equimolar concentrations of PCR product from 92 samples were pooled into a single Illumina lane. Library sequencing was conducted at the Vincent J. Coates Genomic Sequencing Laboratory in the California Institute for Quantitative Biosciences (QB3) at the University of California, Berkeley.

Quantities of airborne fungi in settled dust samplers could be compared because the same time and area of collections were used for all samples. The relative amount of fungal DNA in each settled dust sample was determined using quantitative-PCR (qPCR) with the Bio-Rad CFX96 Touch Real-Time PCR Detection System. qPCR employed ITS primers [30] and SYBR Green. The quantification standard consisted of in-house ITS plasmids that had been constructed from *Aspergillus fumigatus*. Fungal biomass for airborne fungi was estimated by dividing the qPCR fungal gene copy number by the petri dish surface area (56.5cm^2^ for a 10cm plate).

### Sequence processing

Using Cutadapt [31], adapter sequences were removed with no quality filtering, but with a minimum read length of 75bp. Further processing into “amplicon sequence variants” (ASVs) was implemented in the DADA2 library [32] in the R environment with some additional software. First, forward and reverse reads were filtered (truncQ = 2, and maxEE = 2 for forward and maxEE = 5 for reverse reads). Then paired forward and reverse reads were identified using Fastq-pair (https://github.com/linsalrob/EdwardsLab/) and paired using Pear [33]. Returning to DADA2, sequences with N’s were removed, dereplicated, and then sequence variants inferred. Chimeric sequences were removed, and taxonomy assigned against the UNITE database [34].

The two negative controls were clean, containing low total number of reads and low abundance of particular taxa that were abundant in biological samples, indicating likely “sample bleed” [35]. Two low-abundance taxa were identified as contaminants using the Decontam package [36] in R, and these were removed. The mock community contained DNA from 18 taxa, and 28 ASVs were identified. Of the 10 taxa put into the Mock in low concentration, only Cladosporium, Penicillium, and Stachybotrys were recovered. Of the four taxa put in medium relative concentration, all except Candida were recovered. Similarly, for the taxa put in high concentration, only Candida was not recovered. This workflow provided 8,970 ASVs with resolved fungal taxonomic identification, which were used in further analysis. Raw sequences are available through NCBI (SRP144641).

### Statistical analysis

We compared fungal biomass, richness, evenness, and community composition in dust samples from units with visible mold, units with no visible mold, and the outdoors. Statistical analysis of ASVs and quantitative-PCR data was conducted principally in QIIME [37] and R [38] using the Vegan [39], BiodiversityR [40], Phyloseq [41], ggplot2 [42], and Codaseq [43] packages. Gloor et al. [43] argue that microbiome datasets generated by high-throughput sequencing are compositional in nature because the number of DNA sequence reads is limited by the capacity of the sequencing machinery. Thus we analyzed this dataset compositionally by first filtering with CodaSeq (min.reads = 5000, min.occurrence = 0.001, min.prop = 0), then conducting a center log-ratio transformation (clr), instead of using standard counts and rarefying.

We used a one-way ANOVA to analyze qPCR data from settled dust samples and tested for differences in mean fungal biomass across units with no visible mold, units with visible mold, and the outdoors. A Wilcoxon test was used to test for differences in biomass indoors and outdoors, and between units with and without visible mold. Spearman Correlation was used to test whether fungal biomass is significantly different on various floors of the building. ANOVA was used to test whether biomass differed between rooms indoors.

To assess alpha diversity amongst fungal communities in units with or without visible mold as well as outdoors, richness was measured as Chao1 and evenness was measured as Shannon Diversity. Significant differences in means of Chao1 and Shannon between the outdoors, units with visible mold, and units with no visible mold were tested using ANOVA. Spearman Correlation was used to test whether fungal richness is significantly different on various floors of the building, and Kruskal-Wallis was used to test whether richness differed between rooms indoors.

Beta diversity was assessed with Aitchison distance (Euclidian distance between samples) and variance-based compositional principal component (PCA) plots. Two samples were removed as outliers after plotting due to low read counts. Significant differences of fungal community composition in settled dust from units with visible mold, units with no visible mold, and the outdoors was tested using ADONIS (PERMANOVA). We conducted a multivariate homogeneity of groups dispersion test to examine among-community similarity in outdoor samples, units with visible mold, and units with no visible mold, using pairwise permutation tests with Tukey’s HSD. ADONIS was used to test whether fungal composition is significantly different on various floors of the building and rooms indoors.

A Kruskal-Wallis test of median abundance was used to determine whether the abundance of fungal classes were significantly different in outdoor samples, units with no visible mold, and units with visible mold. Finally, for units with visible mold, Kruskal-Wallis testing was used to determine whether the abundance of classes was significantly different across sampling methods (settled dust and surface swabs).

## Results

In our study, we addressed the following questions: a) Does fungal biomass, diversity, or community composition, differ between units with visible mold, units with no visible mold, and the outdoors? b) What taxa dominate units with visible mold? C) Do fungi forming visible colonies on surfaces become airborne and contribute to the indoor air microbiome?

### Fungal biomass

**Fig 2:**
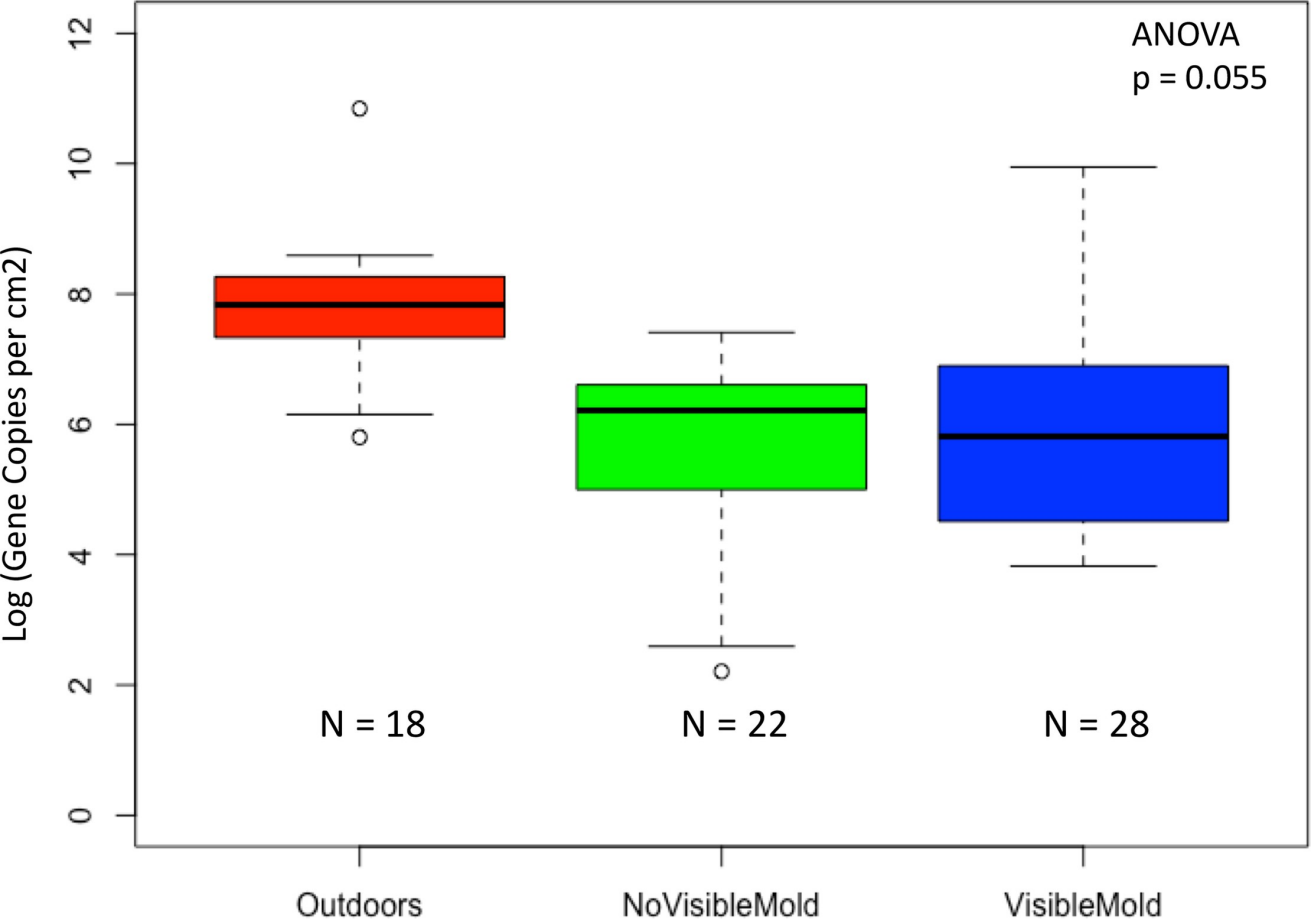
Fungal biomass. Comparison of mean fungal biomass in settled dust from outdoor air (red), indoor air in units with no visible mold (green), and indoor air in units with visible mold (blue). ANOVA was used to compare all three environments. Fungal gene copy number was divided by petri dish surface area as a proxy for biomass per cm^2^ per month. Biomass is log-transformed for visual clarity. Fungal biomass was marginally significantly different between the outdoors, units with no visible mold, and units with visible mold (p = 0.055).

We found that mean fungal biomass was marginally significant (ANOVA; p = 0.055) across indoor units with visible mold, indoor units with no visible mold, and the outdoors (Fig 2). This trend was largely a response to the difference of biomass indoors and outdoors. Biomass outdoors was three times greater than indoors (Wilcoxon; p<0.001). In units with visible mold and units with no visible mold, no significant difference in biomass was detected (Wilcoxon, p = 0.87).

### Fungal diversity

**Fig 3:**
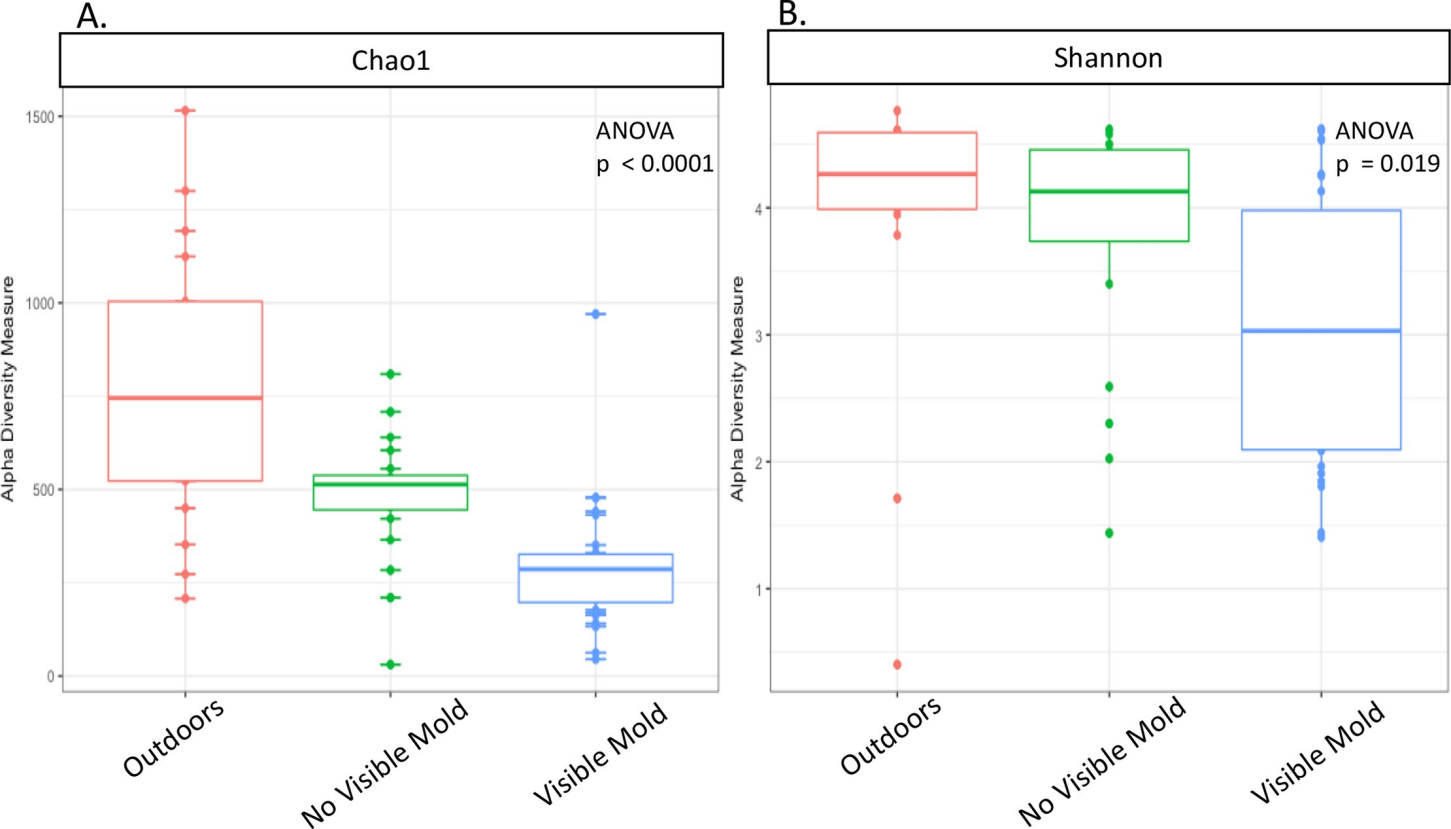
Fungal diversity. Community richness is measured as Chao1 (A) and community evenness is measured with Shannon Diversity Index (B) in settled dust from outdoor air (red), indoor air in units with no visible mold (green), and indoor air in units with visible mold (blue). There are significant differences in community richness (p<0.0001) and evenness (p = 0.019) between these environments. Units with visible mold are less rich and less even than units with no visible mold and the outdoors.

We found significant differences in fungal richness (ANOVA; p<0.001) in settled dust from units with visible mold, units with no visible mold, and the outdoors (Fig 3A). Outdoor samples had the greatest richness, followed by units with no visible mold, and then units with visible mold. Fungal communities in units with visible mold were significantly less even (ANOVA, p = 0.019) than units with no visible mold and the outdoors (Fig 3B).

#### Fungal communities

**Fig 4:**
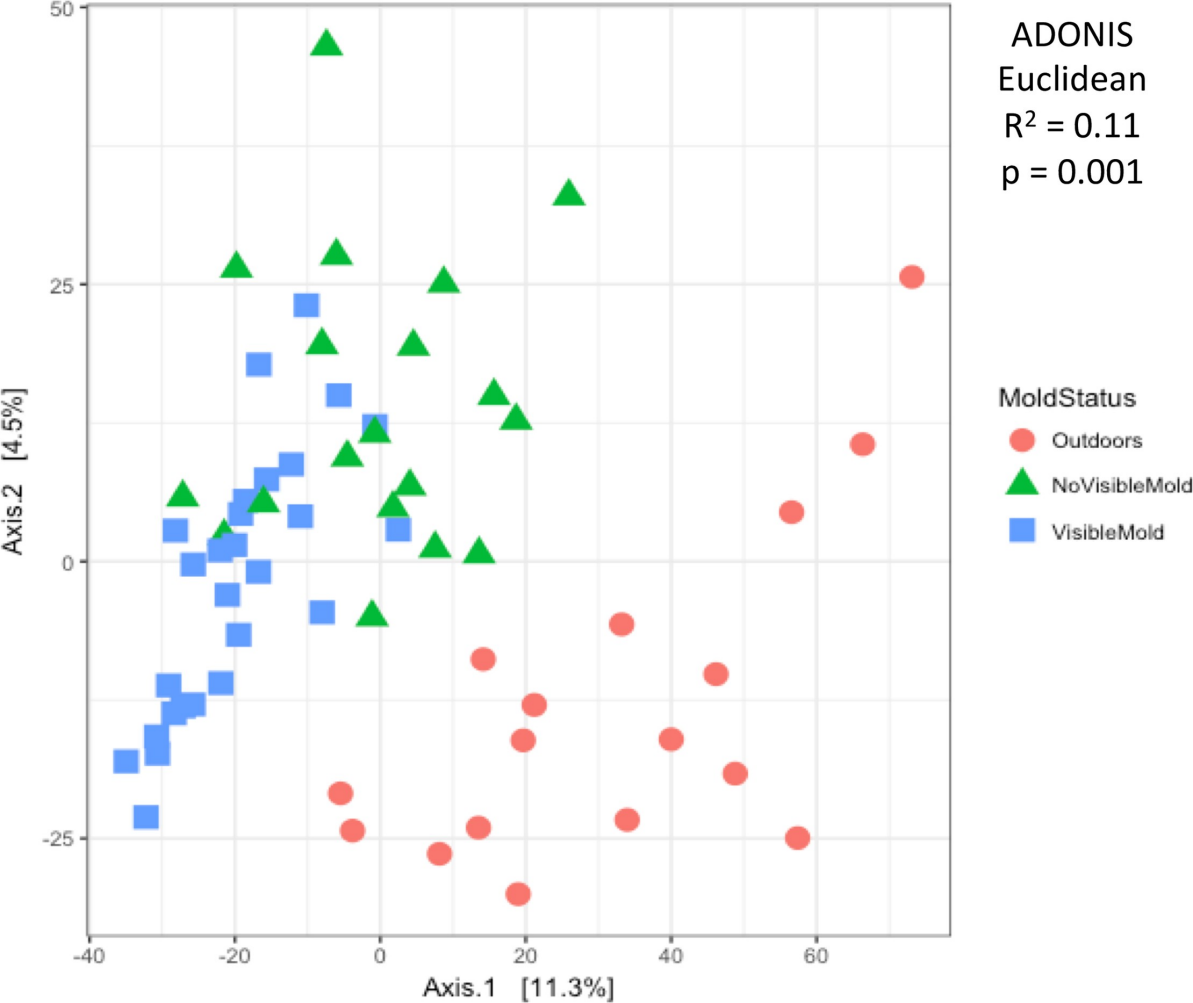
PCA of samples. Variance-based principal components analysis plot showing the dissimilarity of communities in settled dust from outdoor air (red), units with no visible mold (green), and units with visible mold (blue). Outdoor communities are distinct from indoor samples, and the presence of mold indoors distinguishes the composition of indoors samples. ADONIS test shows that the compositional distance between these three environments is significant (p = 0.001; R2 = 0.11).

We found distinct fungal communities outdoors, in units without visible mold, and in units with visible mold (ADONIS; p<0.001; R^2^ = 0.11). Outdoor samples are compositionally distinct from indoor samples, and within indoor samples there are distinct fungal communities in units with visible mold and units with no visible mold (Fig 4). Units with visible mold had the least dispersion within groups, while the outdoor samples showed the greatest dispersion, or greatest dissimilarity to each other (S1 Fig). The distance to centroid of samples from units with visible mold are significantly greater than both units with no visible mold and the outdoors (Tukey HSD; p = 0.02).

### Fungal community structure by rooms and floors

We detected no significant differences in biomass across different floors in the building (Correlation; p = 0.13), or in different rooms indoors (Kruskal-Wallis; p = 0.67). We detected no significant difference in fungal richness across floors (Correlation; p = 0.67), or in different rooms (ANOVA; p = 0.3). We likewise detected no significant differences in fungal community composition across floors (ADONIS; p = 0.26), or in different rooms indoors (ADONIS; p = 0.311).

### Fungal taxa in settled dust

**Fig 5:**
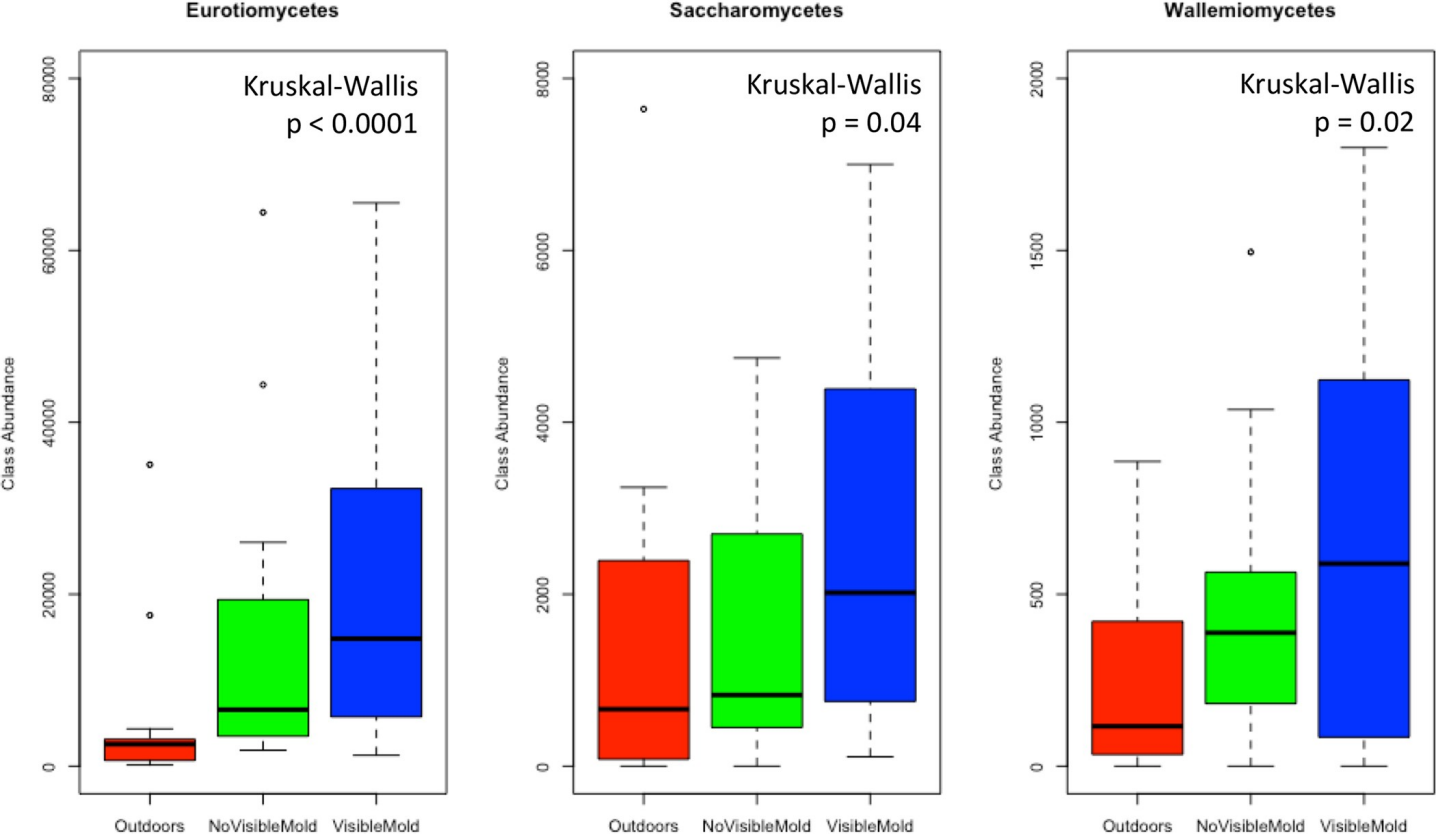
Increased abundance of three classes in units with visible mold. A Kruskal-Wallis test of median abundance determined that three classes, Eurotiomycetes, Saccharomycetes, and Wallemiomycetes, were significantly more abundant in units with visible mold compared to units with no visible mold and the outdoors (p<0.05).

Twelve fungal classes were shown to have significantly different (Kruskal-Wallis; p<0.05) abundances across outdoor samples, units with no visible mold, and units with visible mold (S2 Fig). Agaricomycetes, Agaricostilbomycetes, Arthoniomycetes, Cystobasidiomycetes, Dothideomycetes, Eurotiomycetes, Lecanoromycetes, Leotiomycetes, Pucciniomycetes, Saccharomycetes, Taphrinomycetes, and Wallemiomycetes showed differentially abundant across these three environments. The abundance was significantly greater outdoors in all but four classes. Agaricomycetes, Eurotiomycetes, Saccharomycetes and Wallemiomycetes, had greater abundance indoors compared to the outdoors. Of these, Eurotiomycetes, Saccharomycetes, and Wallemiomycetes, were more abundant in units with visible mold compared to units with no visible mold (Fig 5).

### Visible, surface communities compared to airborne settled dust

**Fig 6:**
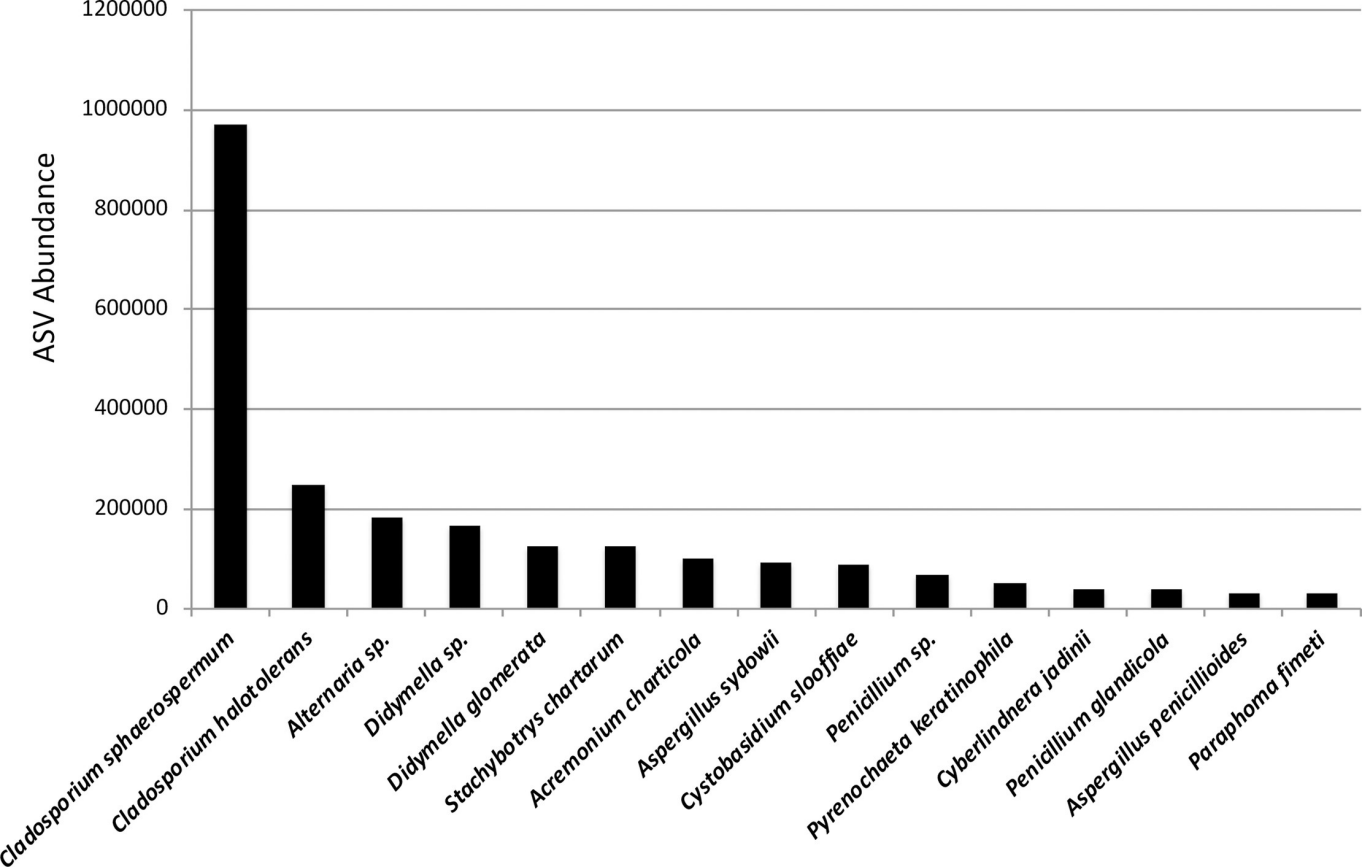
Most abundant taxa in surface samples. The top 15 most abundant taxa recovered in surface samples. Cladosporium spp. comprise the majority of reads from surface samples. Other Dothideomycetes and Eurotiomycetes are major constituents of colonies of visible mold in water-damaged units.

In units with visible mold, swabs were used to collect surface samples. Surface samples were dominated by one major taxon, identified as *Cladosporium sphaerospermum* (Fig 6). Other Dothidiomycetes and Eurotiomycetes were major constituents of surface samples, with *Acremonium*, *Alternaria*, *Aspergillus*, *Cladosporium*, *Cyberlindnera*, *Cystobasidium*, *Didymella*, *Paraphoma*, *Penicillium*, *Pyrenochaeta*, and *Stachybotrys* species comprising the top 15 most abundant taxa in surface samples.

**Fig 7:**
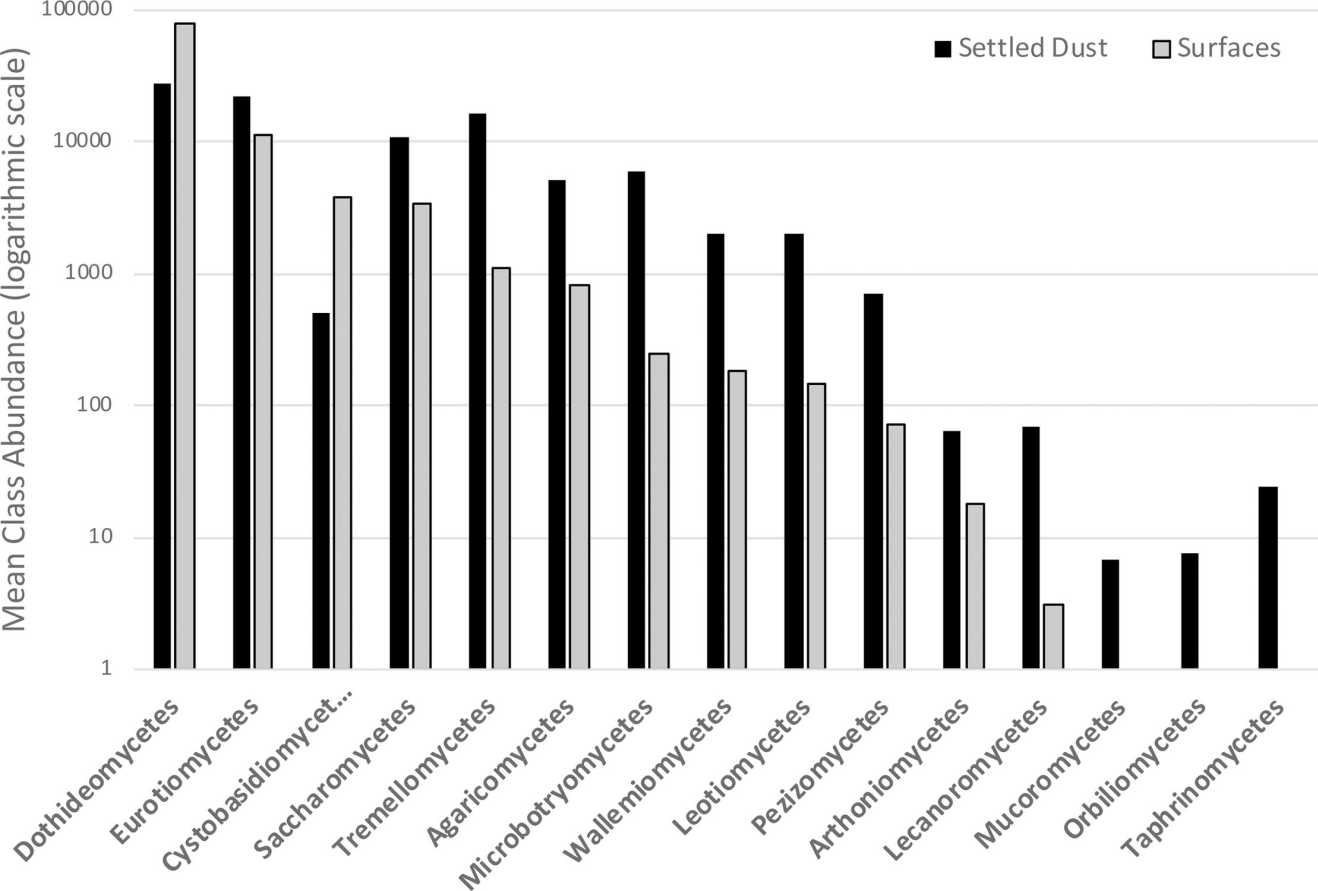
Abundance of fungal classes with differential abundance across sampling methods. 15 classes were found to have significant differential abundance (p<0.05) when sampled using either surface swabs or settled dust collectors in units with visible mold. Settled dust collectors detect greater abundance of fungal classes compared to surface samples in all but two class, Cystobasidiomycetes and Dothideomycetes.

In units with visible mold we also compared the single most abundant taxon in each unit as determined by sampling either with surface swabs or settled dust collectors (S1 Table). For 9 out of 11 units with visible mold, there is discordance between sampling methods for determining which taxon is the most abundant. Surface samples and settled dust collectors detect a different assortment of fungi in units with visible mold. 15 out of 22 classes detected in units with visible mold were found to have significant differential abundance (Kruskal-Wallis; p<0.05) between sampling methods (Fig 7). Settled dust samples detect a wider array of fungal classes than surface samples, and greater abundance of taxa within shared classes. Cystobasidiomycetes and Dothideomycetes were the only classes found to be more abundant in surface samples.

### Comparison of statistical analytical methods

To enable comparison of our treatment of sequence data as compositional with the traditional treatment of such data as abundance counts (as has been done in almost all publications on fungi in indoor air), we also analyzed our data in the traditional manner. Results of both approaches arrive at similar conclusions about the biology of fungi in the built environment. As seen with compositional analysis, count analysis provided an ANOVA of OTU richness that was significantly different (p<0.001) between outdoor samples, units with no visible mold, and units with visible mold (S3 Fig). With count analysis, an NMDS and ADONIS test showed that the composition of units with visible mold were distinct from units with no visible mold and outdoors samples (R2 = 0.14; p<0.0001; R2 = 0.14; S3 Fig). Count analysis found that samples from units with no visible mold were compositionally more similar to outdoor samples, and samples from units with visible mold were distinct from each other and not tightly clustered.

## Discussion

The features that combine to make our study unique in the field of indoor air microbiology include: (1) simultaneous collection indoors and outdoors of fungi passively settling on sterile surfaces over a defined period long enough to account for daily and weekly variation in fungal abundance and occupant behavior. (2) Sampling 21 units within one water-damaged building in residences both with and without visible mold colonies. (3) Culture independent characterization of fungal communities by high-throughput DNA sequencing. (4) Analytical treatment of microbial DNA sequence reads as a compositional dataset rather than the standard statistical treatment of rarefied read counts.

By sequencing settled dust from units with visible mold, units with no visible mold, and outdoor air, we queried the impact of water damage on fungal biomass, richness, and community composition indoors. By sequencing indoor surfaces with visible mold growth, we asked which taxa dominate units with visible mold, and whether these taxa become airborne. With these data we found distinct fungal communities associated with moldy housing. Compared to units with no visible mold and the outdoors, units with visible mold had reduced community richness, reduced community evenness, and a demonstrably different suite of taxa, principally within the Dothideomycetes and Eurotiomycetes. The abundances of Eurotiomcyetes, Saccharomycetes, and Wallemiomyctes were significantly greater in units with visible mold compared to units with no visible mold and the outdoors. Fungal community structure (biomass, richness, and composition) did not differ significantly across floors of the building or between rooms indoors.

A prior study of fungal communities inside healthy homes of the San Francisco Bay Area found that movement of fungi from the outdoors was sufficient to explain fungal assemblages indoors [21]. In our study of a poorly-maintained building in the same region, we saw evidence that the presence of excess water in units, judged by visible mold, allowed for the proliferation of a few indoor taxa and the development of fungal communities that are distinct from those found in units without visible mold or the outdoors.

### Comparison of units with and without visible mold

#### Quantitative analysis did not allow for the detection of differences between units

In this study we found no significant difference of fungal biomass in units with visible mold and units with no visible mold. Fungal biomass was predictably greater outdoors compared to indoors, but qPCR analysis alone was not sufficient to distinguish differences among fungal communities in units with and without visible mold. There is a long history of using quantitative-PCR to ascertain the concentrations of mold indoors (i.e. [44], [45]) such that Mold-Specific Quantitative PCR informed the development of the Environmental Relative Moldiness Index (ERMI), a scoring system used to predict whether dust in buildings can indicate water damage [46].

In comparison to culture-based methods, qPCR is more sensitive, accurate, and better able to detect different microbial concentrations in house dust of moisture-damaged and undamaged homes [47]. Early studies using qPCR suggested that high concentrations of particular fungi indoors (some *Aspergillus*, *Eurotium*, *Chaetomium*, *Paecilomyces*, *Penicillium*, *Scopulariopsis*, *Stachybotrys*, *Trichoderma*, and *Wallemia*) could be indicator species used to detect water damage in homes [44]. In this study we used universal fungal primers to quantify the relative concentrations of all fungi indoors and outdoors, rather than specific taxa, and overall fungal biomass indoors was not significantly different in units either with or without visible mold. Rather, in our study, community composition was more indicative of differences between units than total fungal load. Alpha- and beta-diversity measures allowed for greater detection of differences attributable to water-damage than quantification of fungal gene copies.

#### Fungal communities in units with visible mold are less diverse than units with no visible mold and outdoor air

In our study, units with visible mold had lower fungal richness (number of fungal taxa) than units with no visible mold or outdoor air. In addition, fungal communities in units with visible mold were less even, suggesting a dominance of a few taxa within these units. An assessment of fungal diversity in a water-damaged office building likewise found reduced fungal richness in sequences collected from lower floors that had incurred worse water-damage [48]. But in a study evaluating which housing characteristics impact microbial communities indoors, Kettleson et al. [49] found homes with higher ERMI and high humidity housed more fungal taxa than homes with low ERMI scores and lower humidity. Using clone libraries, Pitkaranta et al. [12], observed elevated fungal diversity was associated with water damage in office buildings. Dannemiller et al. [50] found no significant difference in fungal richness in homes with and without visible mold, but did correlate the presence of water leaks with increased fungal richness. Later Dannemiller et al. [51] showed that in homes with no visible mold, increased moisture led to significantly greater fungal richness, but in homes with visible mold an increase in moisture did not increase fungal richness.

Taken together, these publications suggest that the elevated presence of water indoors may increase fungal richness until a threshold is reached where visible mold becomes present and begins to dominate the community. At that point, actively growing mold may lower observed fungal diversity in water-damaged homes because the airborne spores of a few dominant taxa comprise the majority of sequence data. Such a trend was demonstrated in Adams et al. [52] when a unique signal from abundantly sporulating Basidiomycota fruiting bodies distorted the perception of species richness in mycology classrooms, which appeared to have lower overall richness compared to other classrooms.

#### Fungal communities from units with visible mold are distinct from units with no visible mold and outdoor air

Detecting differences in fungal community composition within homes impacted by water-damage remains challenging, even with the advent of high-throughput sequencing, possibly because there is currently no consistent measurement used to characterize moisture in buildings [53]. In our study we used the appearance of visible mold as the main determinant of water damage in the units, and we were able to detect clear compositional differences in units with and without visible mold. We found that fungal communities in units with visible mold are dissimilar to outdoor air communities and to units with no visible mold. Adams et al. [21], surveyed the microbiota of healthy homes in the Bay Area and found that outdoor air fungi dominate the patterning of indoor air, and no taxa were indicators of the indoor environment. Here we observed the presence or absence of visible mold indoors as a major factor distinguishing the microbial communities within a building. Emerson et al. [26] surveyed homes that had experienced water damage directly attributable to a historic flooding event and found significant differences in fungal community composition between flooded and non-flooded homes in Colorado. Jayaprakash et al. [25] surveyed buildings with less obvious causes of water-damage and did not see fungal community structure differentiate as a response to water intrusion in severely-damaged homes in Finland.

#### Units with visible mold are dominated by fungal taxa previously associated with water damaged buildings

Units with visible mold were dominated by Dothideomycetes in the genera *Alternaria*, *Cladosporium*, *Didymella*, and *Mycosphaerella*, which presumably migrated indoors from the outdoors [21], where their abundance was much greater. The migration of these fungi could also be by air currents or by occupants and their pets [54]. In addition, it is possible that the fungi entered the units on contaminated fruits or vegetables. Three fungal classes, Eurotiomceyetes, Saccharomycetes, and Wallemiomceyetes, showed significantly greater abundance in units with visible mold compared to units with no visible mold and the outdoors, suggesting an indoor source for these taxa in water-damaged units. Agaricomycetes also had greater abundance indoors compared to the outdoors, but their abundance was highest in units with no visible mold. A clone-library study of fungi in dust collected from water-damaged and renovated buildings likewise discovered increased fungal diversity in the Agaricomyetes and Dothideomcyetes fungi was associated with water damage [12].

Many of the taxa that we found to dominate units with visible mold in surface samples have previously been associated with the indoor environment, and in particular water-damaged buildings. The most abundant taxon recovered from colonies of visible mold growth on surfaces was *Cladosporium sphaerospermum*. Other predominant taxa that we recovered in our sequencing of units with visible mold included species in the genera *Acremonium*, *Alternaria*, *Aspergillus*, *Penicillium*, *Stachybotrys*, and *Wallemia*. In a qPCR study of Finnish homes, rising concentrations of *Cladosporium sphaerospermum* and *Wallemia sebi* in house dust were associated with increasing severity of moisture damage [47]. Where we have detected fungal species not previously associated with the indoor environment, the genera or classes that harbor these species have been reported from water-damaged building materials (e.g. in [8], [9], [44], [55]). We identified *Acremonium charticola*, *Aspergillus proliferans*, *Blumeria graminis*, *Cladosporium delicatulum*, *Cladosporium halotolerans*, *Cryptococcus uniguttulatus*, and *Mycosphaerella tassiana*, as dominant taxa in units with visible mold that had not previously been reported indoors.

### Comparison of sampling methods

#### Airborne fungi and surface communities are distinct in units with visible mold

In units with visible mold we collected swabs from actively growing colonies on surfaces, as well as airborne settled dust over the course of one month. The two collection methods provided different profiles of the mycobiota within homes. The dominant taxon recovered within each unit varied between collection methods. Only in two out of eleven units were the same dominant taxon identified using both collection methods. Surface samples were largely dominated by *Cladosporium* spp., while settled dust samples recovered more phylogenetic diversity. Settled dust collectors were able to recover significantly greater abundance of a number of fungal classes: Agaricomycetes, Arthoniomycetes, Lecanoromycetes, Leotiomcyetes, Microbtroymycetes, Pezizomycetes, Saccharomceyes, Tremellomycetes, and Wallemiomycetes, to name a few. These taxa appear to become airborne, and over the course of one month are able to deposit in settled dust collectors. Interestingly, Basidiomycete yeasts in the Cystobasidiomycetes were recovered in greater abundance from surface samples compared to settled dust; possibly suggesting they are less likely to produce airborne spores or be inhaled by residents.

The utilization of varied collection methods provided for a composite view of fungal communities indoors. Collection by swabbing surfaces selects for live fungi that may be actively sporulating in homes at the moment of sampling. These colonies may also be very large in size and have pigmented spores, making them visually detectable. In contrast, settled dust samples are a collection of both live and dead airborne fungi that accumulate in the home over time. Settled dust samples also collect a greater abundance of fungi because they are set out for weeks at a time and can acquire fungal material from undetected colonies as well. The settled dust collection captures longer-term dynamics in the fungal community composition, while the surface sample is a snapshot of predominant taxa in the unit at a particular moment in time. Temporal, ecological, and presumable physiological differences are detected by different sampling methods, and both proved valuable to characterize fungi associated with water-damaged buildings.

### Limitations of this study

We collected 68 settled dust samples from outdoor air, units with no visible mold, and units with visible mold, within a chronically water-damaged 150-unit building in the San Francisco Bay Area. Although some units had visibly greater water-damage than others (demonstrated by actively growing mold on surfaces), it is possible that all units in the building experienced some level of water damage due to long-term structural issues, and thus we may actually have compared mildly and severely water-damaged units. A more comprehensive assessment of building water intrusion, including the measurement of relative humidity, temperature, and the surface area of mold, might have facilitated a more precise categorization of the level of water damage in each unit. It would have been ideal to have also surveyed a building of similar design and age with no prior history of water-damage in close proximity to our building. This survey would allow for the comparison of microbial communities within a water-damaged building and a healthy building within the same timescale, season, and weather regime. Additionally, surface samples were only collected from units with visible mold where we swabbed actively growing colonies. Though we presumed surface samples would be dominated by one taxon, sequence data recovered additional ASVs in surface samples at lower abundance. These additional taxa are constituents of the surface community that were not necessarily visible. It would be good to standardize surface collections in each unit and have background knowledge of which ASVs are found on surfaces in each unit regardless of the presence of visible mold.

## Conclusions

This study is the first to analyze the microbial inhabitants of a condemned building using high-throughput sequencing methods. This is also one of only a handful of studies to use culture-independent techniques to explore the impact of water-damage on microbial communities in buildings. The distinction between outdoor microbial communities, units with no visible mold, and units with visible mold, shows that insufficient building maintenance can drastically shift the assemblage of fungi indoors.

In this study we showed that sampling replicated units in one poorly maintained structure can reveal differences among the airborne mycobiome seen outdoors and indoors, as well as in units with and without visible fungal colonies. Furthermore, sampling fungal spores, yeast cells, and hyphae, by gravity settling over a time period long enough to account for daily variation in airborne fungi and weekly variation in occupant behavior characterized airborne fungal communities that correlated with the presence of visible fungal colonies. Biomass of the settled fungi, however, did not correlate with the presence or absence of visible fungi in units. In units with visible mold, the airborne fungal communities were less diverse and dominated by a few major taxa.

With the onset of high-throughput sequencing, it is no longer “impractical to measure all the molds in a home” collected in dust, as was suggested by Vesper et al. [46]. We look forward to comparing our study with others of poorly maintained buildings that include replication in the form of many units in one building, sampling of airborne fungi over a defined period, and fungal identification by high-throughput sequencing. Through these comparisons we hope to arrive at an economical and accurate means to detect progress toward the WHO mandate of healthy housing as a basic human right.

## Supporting information

S1 Fig Among-community beta-diversity across environments. Comparison of homogeneity of variance of communities in settled dust sampled from outdoor air (red), indoor air of units with no visible mold (green), and indoor air of units with visible mold (blue). Significantly less dissimilarity (p = 0.02) is seen among communities sampled from units with visible mold compared to those sampled in units without visible mold or the outdoors (Tukey’s HSD test).
(PDF)

S2 Fig Abundance of fungal classes across environments. Sequence abundance of fungal classes found in settled dust from outdoor air (top panel), units with no visible mold (middle), and units with visible mold (bottom). These twelve classes have significantly different abundance across the environments, as determined by Kruskal-Wallis test (p<0.05). Eight classes are more abundant outdoors, but Agaricomycetes, Eurotiomycetes, Saccharomycetes, and Wallemiomycetes are more abundant indoors.
(PDF)

S3 Fig Analysis using traditional count-based methods. Alpha- and beta-diversity of fungal communities in samples when analyzed by traditional count-based methods. (A) Alpha-diversity showing significant differences in OTU richness among all sample types, outdoor air (red), indoor air of units with no visible mold (green), and indoor air of units with visible mold (blue). (B) Beta-diversity showing significant differences in community composition among environments. Comparison of these results with those from analyses that treat sequence data as compositional (Figs 3 and 4) show similar trends in richness and community composition.
(PDF)

S1 Table Predominant taxon in each unit. Table showing which ASV was identified as the most abundant taxon in each unit with visible mold, either by surface samples or settled dust collectors. There is discordance in what taxon predominates each unit depending on sampling method used to survey the community. Stars denote taxa that have not previously been reported from the indoor environment or water damaged buildings.
(PDF)

## References

[1] Klepeis N, Nelson W, Orr W, Robinson J, Tsang A, Switzer P, et al The National Human Activity Pattern Survey (NHAPS): a resource for assessing exposure to environmental pollutants. Journal of Exposure Analysis and Environmental Epidemiology. 2001;11: 231–252. 10.1038/sj.jea.7500165 11477521

[2] Rauh V, Landrigan P, Claudio L. Housing and Health. Annals of the New York Academy of Sciences. 2008;: 1–14. 10.1196/annals.1425.032 18579887

[3] Krieger J, Higgins D. Housing and Health: Time Again for Public Health Action. Research and Practice. 2002;: 1–11. 10.2105/ajph.92.5.758 PMC1447157 11988443

[4] Center for Disease Control. CDC Health Disparities and Inequalities Report—United States, 2011. 2011;: 1–116.

[5] World Health Organization. WHO Guidelines for Indoor Air Quality: Dampness and Mould. 2009;: 1–248. 23785740

[6] Adamkiewicz G, Zota A, Fabia P, Chahine T, Julien R, Spenger J, et al Moving Environmental Justice Indoors: Understanding Structural Influences on Residential Exposure Patterns in Low-Income Communities. Research and Practice. 2011;: 1–9. 10.2105/AJPH.2011.300119 PMC3222513 21836112

[7] Health NYCDO, Hygiene M. Guidelines on Assessment and Remediation of Fungi in Indoor Environments. 2008;: 1–25.

[8] Flannigan B, Samson RA, Miller DJ. Microorganisms in Home and Indoor Work Environments. 2nd ed 2011 pp. 1–511.

[9] Andersen B, Frisvad JC, Søndergaard I, Rasmussen IS, Larsen LS. Associations between Fungal Species and Water-Damaged Building Materials. Appl Environ Microbiol. Sixth edition. 2011;77: 4180–4188. 10.1128/AEM.02513-10 21531835 PMC3131638

[10] Nielsen KF, Frisvad JC. Mycotoxins on building materials Fundamentals of mold growth in indoor environments and strategies for healthy living. Wageningen: Wageningen Academic Publishers; 2011 pp. 245–275. 10.3920/978-90-8686-722-6_9

[11] Rao CY, Riggs MA, Chew GL, Muilenberg ML, Thorne PS, Van Sickle D, et al Characterization of Airborne Molds, Endotoxins, and Glucans in Homes in New Orleans after Hurricanes Katrina and Rita. Appl Environ Microbiol. 2007;73: 1630–1634. 10.1128/AEM.01973-06 17209066 PMC1828784

[12] Pitkäranta M, Meklin T, Hyvärinen A, Nevalainen A, Paulin L, Auvinen P, et al Molecular profiling of fungal communities in moisture damaged buildings before and after remediation—a comparison of culture-dependent and culture-independent methods. BMC Microbiol. 2011;11: 235–16. 10.1186/1471-2180-11-235 22017920 PMC3206440

[13] Amann RI, Ludwig W, Schleifer K-H. Phylogenetic Identification and In Situ Detection of Individual Microbial Cells without Cultivation. Microbiological Reviews. 1995;59: 1–33. 7535888 10.1128/mr.59.1.143-169.1995 PMC239358

[14] Stephens B. What Have We Learned about the Microbiomes of Indoor Environments? Gibbons SM, editor. mSystems. 2016;1: 202–9. 10.1128/mSystems.00083-16 27822547 PMC5069963

[15] Adams RI, Bhangar S, Dannemiller KC, Eisen JA, Fierer N, Gilbert JA, et al Ten questions concerning the microbiomes of buildings. Building and Environment. Elsevier Ltd; 2016;109: 224–234. 10.1016/j.buildenv.2016.09.001

[16] Amend AS, Seifert KA, Samson R, Bruns TD. Indoor fungal composition is geographically patterned and more diverse in temperate zones than in the tropics. Proc Natl Acad Sci USA. 2010;107: 13748–13753. 10.1073/pnas.1000454107 20616017 PMC2922287

[17] Adams RI, Miletto M, Lindow SE, Taylor JW, Bruns TD. Airborne Bacterial Communities in Residences: Similarities and Differences with Fungi. Moreau CS, editor. PLoS ONE. 2014;9: e91283–7. 10.1371/journal.pone.0091283 24603548 PMC3946336

[18] Dannemiller KC, Gent JF, Leaderer BP, Peccia J. Influence of housing characteristics on bacterial and fungal communities in homes of asthmatic children. Indoor Air. 2015;26: 179–192. 10.1111/ina.12205 25833176 PMC4591094

[19] Kembel SW, Jones E, Kline J, Northcutt D, Stenson J, Womack AM, et al Architectural design influences the diversity and structure of the built environment microbiome. The ISME Journal. Nature Publishing Group; 2012;6: 1469–1479. 10.1038/ismej.2011.211 22278670 PMC3400407

[20] Meadow JF, Altrichter AE, Bateman AC, Stenson J, Brown GZ, Green JL, et al Humans differ in their personal microbial cloud. PeerJ. 2015;3: e1258–22. 10.7717/peerj.1258 26417541 PMC4582947

[21] Adams RI, Miletto M, Taylor JW, Bruns TD. Dispersal in microbes: fungi in indoor air are dominated by outdoor air and show dispersal limitation at short distances. The ISME Journal. 2013;7: 1262–1273. 10.1038/ismej.2013.28 23426013 PMC3695294

[22] Barberán A, Dunn RR, Reich BJ, Pacifici K, Laber EB, Menninger HL, et al The ecology of microscopic life in household dust. Proc R Soc B. 2015;282: 20151139–9. 10.1098/rspb.2015.1139 26311665 PMC4571696

[23] Rintala H, Pitkäranta M, Täubel M. Microbial Communities Associated with House Dust. Advances in Applied Microbiology. Elsevier; 2012 pp. 75–120. 10.1016/B978-0-12-394805-2.00004-X 22305094

[24] Adams RI, Tian Y, Taylor JW, Bruns TD, Hyvärinen A, Täubel M. Passive dust collectors for assessing airborne microbial material. Microbiome. Microbiome; 2015;3: 1–11. 10.1186/s40168-014-0066-1 26434807 PMC4593205

[25] Jayaprakash B, Adams RI, Kirjavainen P, Karvonen A, Vepsäläinen A, Valkonen M, et al Indoor microbiota in severely moisture damaged homes and the impact of interventions. Microbiome. Microbiome; 2017;: 1–17. 10.1186/s40168-016-0209-7 29029638 PMC5640920

[26] Emerson JB, Keady PB, Brewer TE, Clements N, Morgan EE, Awerbuch J, et al Impacts of Flood Damage on Airborne Bacteria and Fungi in Homes after the 2013 Colorado Front Range Flood. Environ Sci Technol. 2015;49: 2675–2684. 10.1021/es503845j 25643125

[27] Committee on Microbiomes of the Built Environment: From Research to Application, Board on Life Sciences, Board on Environmental Studies and Toxicology, Division on Earth and Life Studies, Health and Medicine Division, Board on Infrastructure and the Constructed Environment, et al Microbiomes of the Built Environment. Washington, D.C: National Academies Press; 2017 pp. 1–317. 10.17226/23647

[28] White TJ, Bruns T, Lee S, Taylor J. Amplification and direct sequencing of fungal ribosomal rna genes for phylogenetics PCR Protocols: a Guide to Methods and Applications. Academic Press, Inc; 1990 pp. 315–322. 10.1016/B978-0-12-372180-8.50042-1

[29] Adams RI, Lymperopoulou DS, Misztal PK, De Cassia Pessotti R, Behie SW, Tian Y, et al Microbes and associated soluble and volatile chemicals on periodically wet household surfaces. Microbiome. Microbiome; 2017;: 1–16. 10.1186/s40168-016-0209-7 28950891 PMC5615633

[30] Zhou G, Whong W-Z, Ong T, Chen B. Development of a fungus-specific PCR assay for detecting low-level fungi in an indoor environment. Molecular and Cellular Probes. 2000;14: 339–348. 10.1006/mcpr.2000.0324 11090263

[31] Martin M. Cutadapt Removes Adapter Sequences from High-throughput Sequencing Reads. EMBnetjournal. 2011;: 1–3.

[32] Callahan BJ, McMurdie PJ, Rosen MJ, Han AW, Johnson AJA, Holmes SP. DADA2: High-resolution sample inference from Illumina amplicon data. Nat Methods. 2016;13: 581–583. 10.1038/nmeth.3869 27214047 PMC4927377

[33] Zhang J, Kobert K, Flouri T, Stamatakis A. PEAR: a fast and accurate Illumina Paired-End reAd mergeR. Bioinformatics. 2014;30: 614–620. 10.1093/bioinformatics/btt593 24142950 PMC3933873

[34] Abarenkov K, Nilsson RH, Larsson K-H, Alexander IJ, Eberhardt U, Erland S, et al The UNITE database for molecular identification of fungi–recent updates and future perspectives. The New Phytologist. 2010;: 1–35. 10.1111/j.1469-8137.2009.03160.x 20409185

[35] Carlsen T, Aas AB, Lindner D, Vralstad T, Schumacher T, Kauserud H. Don't make a mista(g)ke: is tag switching an overlooked source of error in amplicon pyrosequencing studies? Fungal Ecology. Elsevier Ltd; 2012;5: 747–749. 10.1016/j.funeco.2012.06.003

[36] Davis NM, Proctor D, Holmes SP, Relman DA, Callahan BJ. Simple statistical identification and removal of contaminant sequences in marker-gene and metagenomics data. 2017;: 1–38. 10.1101/221499 PMC6298009 30558668

[37] Caporaso JG, Kuczynski J, Stombaugh J, Bittinger K, Bushman FD, Costello EK, et al QIIME allows analysis of high- throughput community sequencing data. Nature Publishing Group. Nature Publishing Group; 2010;7: 335–336. 10.1038/nmeth0510-335 PMC3156573 20383131

[38] Ihaka R, Gentleman R. R: A Language for Data Analysis and Graphics. Journal of Computational and Graphical Statistics. 1996;5: 299–314. 10.1080/10618600.1996.10474713

[39] Oksanen J, Blanchet FG, Friendly M, Kindt R, Legendre P, McGlinn D, et al Package “vegan.” 2018;: 1–297.

[40] Kindt R, Coe R. Tree diversity analysis: A Manual and Software for Common Statistical Methods for Ecological and Biodiversity Studies. World Agroforestry Center 2005;: 1–7.

[41] McMurdie PJ, Holmes S. phyloseq: An R Package for Reproducible Interactive Analysis and Graphics of Microbiome Census Data. Watson M, editor. PLoS ONE. Public Library of Science; 2013;8: e61217–11. 10.1371/journal.pone.0061217 23630581 PMC3632530

[42] Wickham H. ggplot2. 2009 pp. 1–211.

[43] Gloor GB, Wu JR, Pawlowsky-Glahn V, Egozcue JJ. It's all relative: analyzing microbiome data as compositions. Annals of Epidemiology. Elsevier Inc; 2016;26: 322–329. 10.1016/j.annepidem.2016.03.003 27143475

[44] Vesper SJ, Wymer LJ, Meklin T, Varma M, Stott R, Richardson M, et al Comparison of populations of mould species in homes in the UK and USA using mould-specific quantitative PCR. Letters in Applied Microbiology. 2005;41: 367–373. 10.1111/j.1472-765X.2005.01764.x 16162146

[45] Meklin T, Reponen T, McKinstry C, Cho S-H, Grinshpun S, Nevalainen A, et al Comparison of mold concentrations quantified by MSQPCR in indoor and outdoor air sampled simultaneously. Science Total Environment. 2007;: 1–6. 10.1016/j.scitotenv.2007.03.031 PMC2233941 17467772

[46] Vesper S, McKinstry C, Haugland R, Wymer L, Bradham K, Ashley P, et al Development of an Environmental Relative Moldiness Index for US Homes. Journal of Occupational and Environmental Medicine. 2007;49: 829–833. 10.1097/JOM.0b013e3181255e98 17693779

[47] Lignell U, Meklin T, Rintala H, Hyvärinen A, Vepsäläinen A, Pekkanen J, et al Evaluation of quantitative PCR and culture methods for detection of house dust fungi and streptomycetes in relation to moisture damage of the house. Letters in Applied Microbiology. 2008;47: 303–308. 10.1111/j.1472-765X.2008.02431.x 19241524

[48] Green BJ, Lemons AR, Park Y, Cox-Ganser JM, Park J-H. Assessment of fungal diversity in a water- damaged office building. Journal of Occupational and Environmental Hygiene. Taylor & Francis; 2017;14: 285–293. 10.1080/15459624.2016.1252044 27786737 PMC6314010

[49] Kettleson EM, Adhikari A, Vesper S, Coombs K, Indugula R, Reponen T. Key determinants of the fungal and bacterial microbiomes in homes. Environmental Research. Elsevier; 2015;138: 130–135. 10.1016/j.envres.2015.02.003 25707017 PMC4385485

[50] Dannemiller KC, Mendell MJ, Macher JM, Kumagai K, Bradman A, Holland N, et al Next-generation DNA sequencing reveals that low fungal diversity in house dust is associated with childhood asthma development. Indoor Air. 2014;24: 236–247. 10.1111/ina.12072 24883433 PMC4048861

[51] Dannemiller KC, Gent JF, Leaderer BP, Peccia J. Indoor microbial communities: Influence on asthma severity in atopic and nonatopic children. The Journal of Allergy and Clinical Immunology. Elsevier Ltd; 2016;: 1–9. 10.1016/j.jaip.2015.10.003 26851966 PMC5357886

[52] Adams RI, Amend AS, Taylor JW, Bruns TD. A Unique Signal Distorts the Perception of Species Richness and Composition in High-Throughput Sequencing Surveys of Microbial Communities: a Case Study of Fungi in Indoor Dust. Microb Ecol. 2013;66: 735–741. 10.1007/s00248-013-0266-4 23880792 PMC3824195

[53] Dedesko S, Siegel JA. Moisture parameters and fungal communities associated with gypsum drywall in buildings. Microbiome. Microbiome; 2015;: 1–15. 10.1186/s40168-014-0066-1 26642923 PMC4672539

[54] Adams RI, Bhangar S, Pasut W, Arens EA, Taylor JW, Lindow SE, et al Chamber Bioaerosol Study: Outdoor Air and Human Occupants as Sources of Indoor Airborne Microbes. Zhang Y, editor. PLoS ONE. 2015;10: e0128022–18. 10.1371/journal.pone.0128022 26024222 PMC4449033

[55] Westerdijk Institute Indoor Fungi Database. http://www.westerdijkinstitute.nl/Indoor/Biolomics.aspx Utrecht, The Netherlands.

