# Supplementary figures and images for: A different suite: The assemblage of distinct fungal communities in water-damaged units of a poorly-maintained public housing building
Source: PLoS One. 2019 Mar 18;14(3):e0213355. doi: 10.1371/journal.pone.0213355 (PMC6422403; doi:10.1371/journal.pone.0213355)

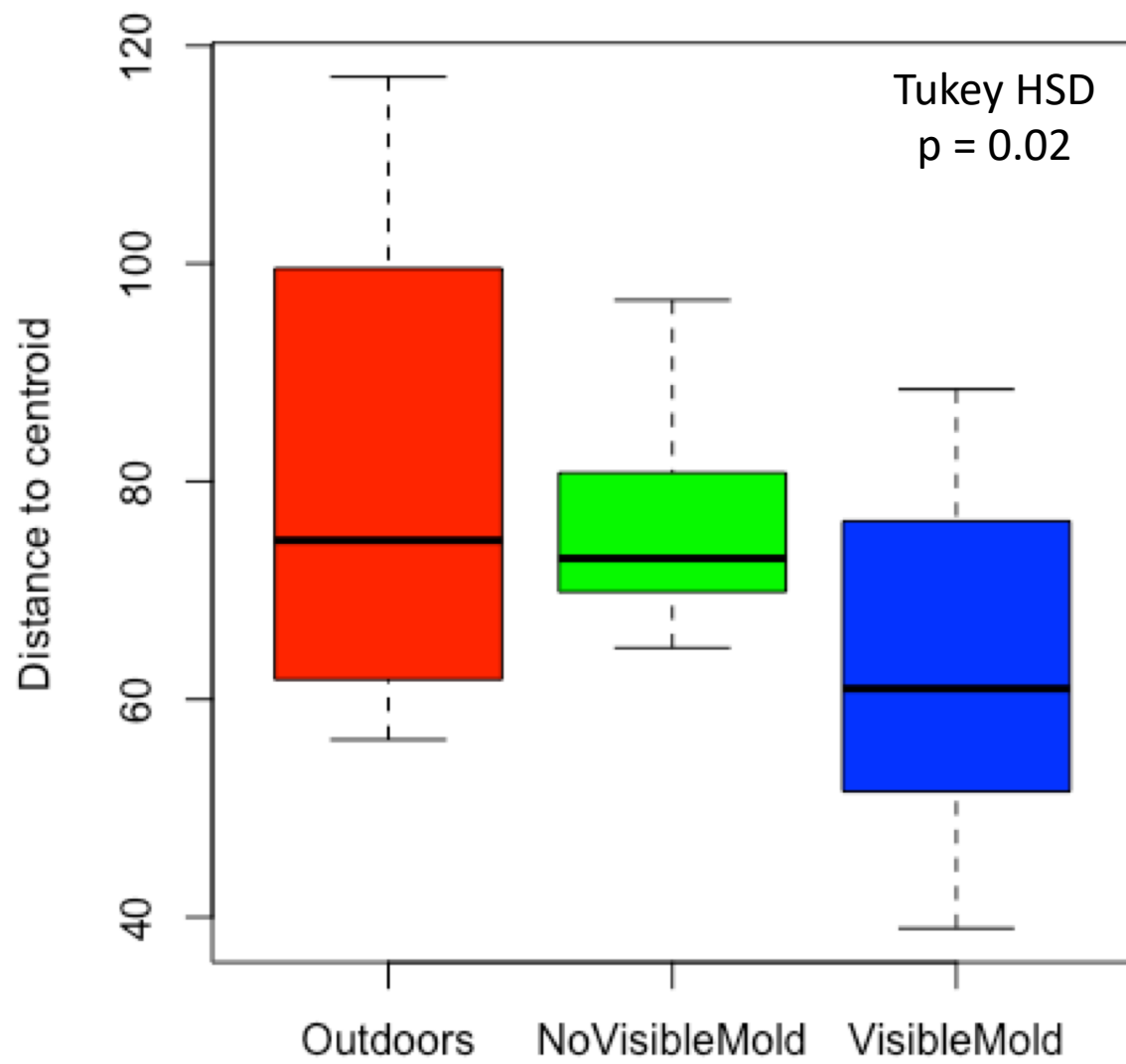

Supplement: S1 Fig — Comparison of homogeneity of variance of communities in settled dust sampled from outdoor air (red), indoor air of units with no visible mold (green), and indoor air of units with visible mold (blue). Significantly less dissimilarity (p = 0.02) is seen among communities sampled from units with visible mold compared to those sampled in units without visible mold or the outdoors (Tukey’s HSD test). (PDF) [file pone.0213355.s001.pdf]

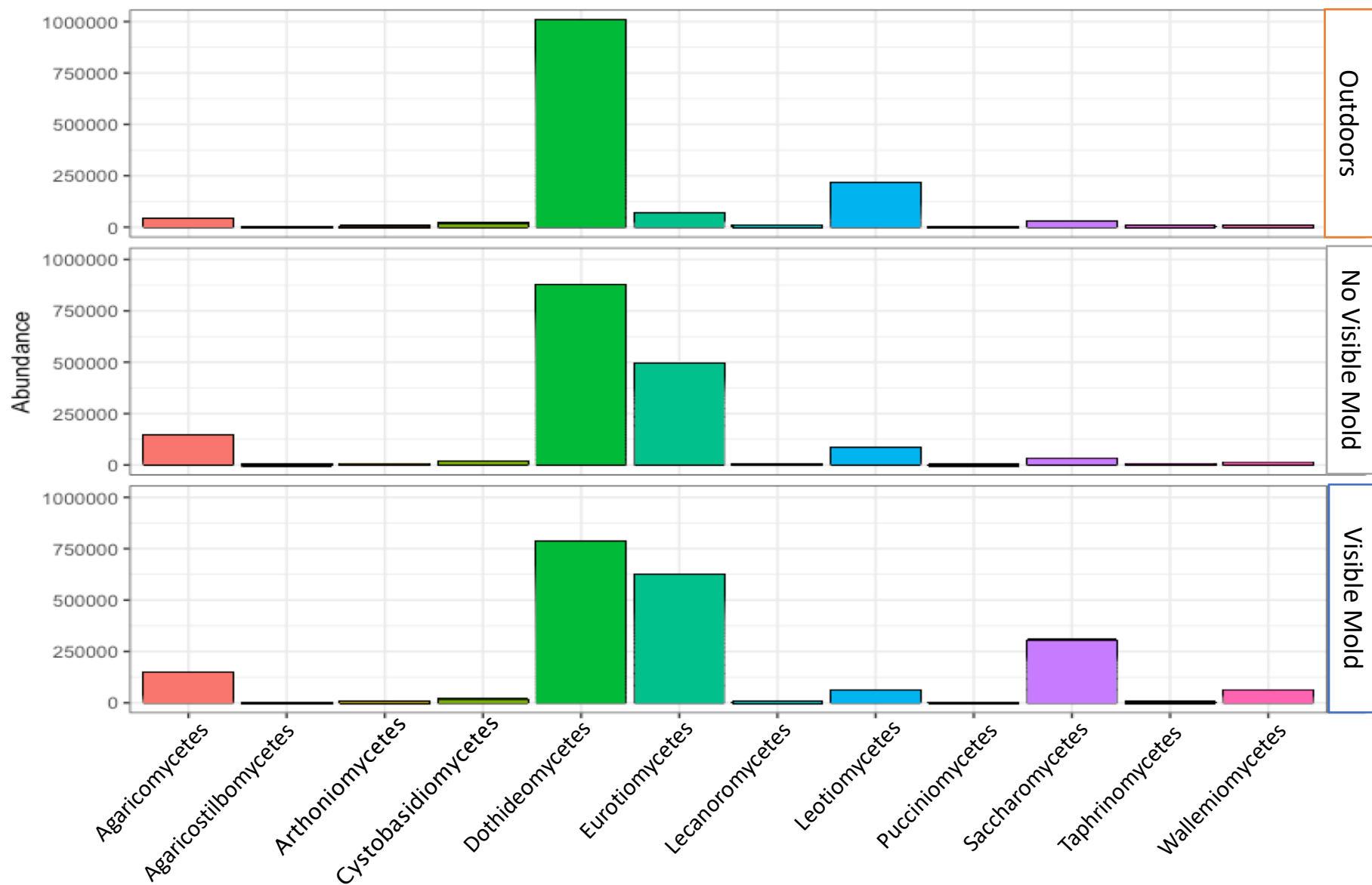

Supplement: S2 Fig — Sequence abundance of fungal classes found in settled dust from outdoor air (top panel), units with no visible mold (middle), and units with visible mold (bottom). These twelve classes have significantly different abundance across the environments, as determined by Kruskal-Wallis test (p<0.05). Eight classes are more abundant outdoors, but Agaricomycetes, Eurotiomycetes, Saccharomycetes, and Wallemiomycetes are more abundant indoors. (PDF) [file pone.0213355.s002.pdf]

## A. Alpha-Diversity

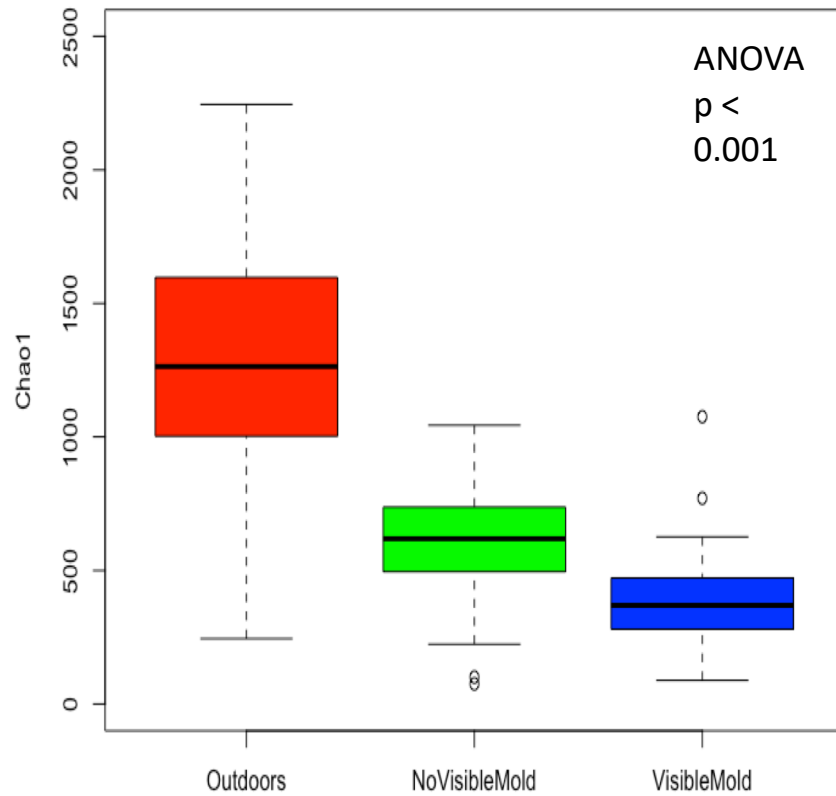

## B. Beta-Diversity

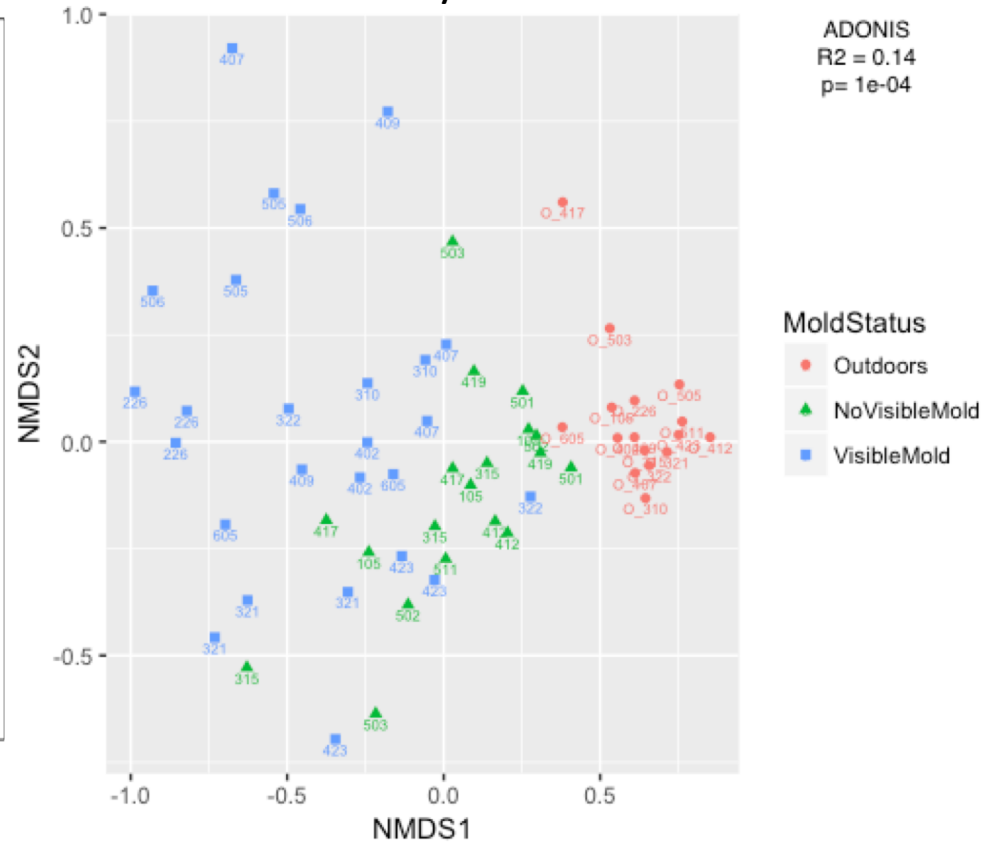

Supplement: S1 Table — Table showing which ASV was identified as the most abundant taxon in each unit with visible mold, either by surface samples or settled dust collectors. There is discordance in what taxon predominates each unit depending on sampling method used to survey the community. Stars denote taxa that have not previously been reported from the indoor environment or water damaged buildings. (PDF) [file pone.0213355.s004.pdf]
