# Supplementary material for: A different suite: The assemblage of distinct fungal communities in water-damaged units of a poorly-maintained public housing building
Source: PLoS One. 2019 Mar 18;14(3):e0213355. doi: 10.1371/journal.pone.0213355 (PMC6422403; doi:10.1371/journal.pone.0213355)
Supplement: S3 Fig — Alpha- and beta-diversity of fungal communities in samples when analyzed by traditional count-based methods. (A) Alpha-diversity showing significant differences in OTU richness among all sample types, outdoor air (red), indoor air of units with no visible mold (green), and indoor air of units with visible mold (blue). (B) Beta-diversity showing significant differences in community composition among environments. Comparison of these results with those from analyses that treat sequence data as compositional (Figs 3 and 4) show similar trends in richness and community composition. (PDF) [file pone.0213355.s003.pdf]

Unit	Most Dominant Taxon in Settled Dust	Most Dominant Taxon on Surfaces
A	* *Aspergillus proliferans* | Eurotiomycetes	*Cladosporium sphaerospermum* | Dothideomycetes
B	* *Mycosphaerella tassiana* | Dothideomycetes	*Aspergillus sydowii* | Eurotiomycetes
C	* *Cladosporium delicatulum* | Dothideomycetes	* *Mycosphaerella tassiana* | Dothideomycetes
D	*Clavispora lusitaniae* | Saccharomycetes	* *Cryptococcus uniguttulatus* (syn. *C. neoformans*) | Tremellomycetes
E	* *Blumeria graminis* | Leotiomycetes	*Cladosporium sphaerospermum* | Dothideomycetes
F	*Aspergillus sydowii* | Eurotiomycetes	*Cyberlindnera jadinii* (syn. *Pichia jadinii*) | Saccharomycetes
G	*Aspergillus sydowii* | Eurotiomycetes	*Aspergillus sydowii* | Eurotiomycetes
H	* *Mycosphaerella tassiana* | Dothideomycetes	*Aspergillus sydowii* | Eurotiomycetes
I	*Penicillium sp.* | Eurotiomycetes	*Didymella sp.* | Dothideomycetes
J	* *Acremonium charticola* | Sordariomycetes	* *Cladosporium halotolerans* | Dothideomycetes
K	*Alternaria sp.* | Dothideomycetes	*Alternaria sp.* | Dothideomycetes

* Denote taxa that have previously not been reported in indoor environments
